# Perfluorocarbon Particle Size Influences Magnetic Resonance Signal and Immunological Properties of Dendritic Cells

**DOI:** 10.1371/journal.pone.0021981

**Published:** 2011-07-19

**Authors:** Helmar Waiczies, Stefano Lepore, Nicole Janitzek, Ulrike Hagen, Frank Seifert, Bernd Ittermann, Bettina Purfürst, Antonio Pezzutto, Friedemann Paul, Thoralf Niendorf, Sonia Waiczies

**Affiliations:** 1 Experimental and Clinical Research Center, Max Delbrück Center for Molecular Medicine and Charité Medical Faculty, Berlin, Germany; 2 Berlin Ultrahigh Field Facility, Max-Delbrück Center for Molecular Medicine, Berlin, Germany; 3 Physikalisch Technische Bundesanstalt, Berlin, Germany; 4 Molecular Immunotherapy, Max Delbrück Center for Molecular Medicine, Berlin, Germany; 5 Electron Microscopy, Max Delbrück Center for Molecular Medicine, Berlin, Germany; 6 Department of Hematology and Oncology, Charité, Berlin, Germany; 7 NeuroCure Clinical Research Center, Charité, Berlin, Germany; 8 Clinical and Experimental Multiple Sclerosis Research Center, Charité, Berlin, Germany; 9 Department of Anatomy, University of Malta, Msida, Malta; Julius-Maximilians-Universität Würzburg, Germany

## Abstract

The development of cellular tracking by fluorine (^19^F) magnetic resonance imaging (MRI) has introduced a number of advantages for following immune cell therapies *in vivo*. These include improved signal selectivity and a possibility to correlate cells labeled with fluorine-rich particles with conventional anatomic proton (^1^H) imaging. While the optimization of the cellular labeling method is clearly important, the impact of labeling on cellular dynamics should be kept in mind. We show by ^19^F MR spectroscopy (MRS) that the efficiency in labeling cells of the murine immune system (dendritic cells) by perfluoro-15-crown-5-ether (PFCE) particles increases with increasing particle size (560>365>245>130 nm). Dendritic cells (DC) are professional antigen presenting cells and with respect to impact of PFCE particles on DC function, we observed that markers of maturation for these cells (CD80, CD86) were also significantly elevated following labeling with larger PFCE particles (560 nm). When labeled with these larger particles that also gave an optimal signal in MRS, DC presented whole antigen more robustly to CD8+ T cells than control cells. Our data suggest that increasing particle size is one important feature for optimizing cell labeling by PFCE particles, but may also present possible pitfalls such as alteration of the immunological status of these cells. Therefore depending on the clinical scenario in which the *^19^*F-labeled cellular vaccines will be applied (cancer, autoimmune disease, transplantation), it will be interesting to monitor the fate of these cells *in vivo* in the relevant preclinical mouse models.

## Introduction

The prospect of being able to non-invasively track immune cells *in vivo* is not only fascinating for scientists studying the pathogenesis of autoimmune conditions such as multiple sclerosis or rheumatoid arthritis but also for clinical researchers administering immune cell therapies such as dendritic cell vaccines in cancer trials [1]. One key consideration that is often neglected in clinical studies dealing with cellular therapy is the rate of cell delivery and distribution, which highly depends on the fate and function of the transplanted cells. Originally, clinically applied cells such as dendritic cells (DC) were monitored using scintigraphic scanning with radioactive ^111^In [2], although one major drawback with this imaging technique is the lack of anatomical detail. Cellular tracking in combination with ^1^H magnetic resonance imaging (MRI) overcomes this drawback. Proton (^1^H) MRI employs spin physics to determine water content and therefore properties of tissue which makes this technique valuable to uncover anatomical as well as any underlying pathophysiological details. Cellular MRI has commonly been performed using T2*-weighted ^1^H imaging with superparamagnetic small iron-oxide (SPIO) nanoparticles [3].

Non-invasive tracking of cells by MRI should prove to be an important complement within the fields of cancer, autoimmunity and transplantation medicine. However, the available susceptibility contrast agents such as the SPIO nanoparticles often make it difficult for MRI scientists to differentiate the labeled cellular transplants from other susceptibility-related T2* effects such as paramagnetic deoxygenated blood [4]. An MRI technology using fluorine (^19^F)-rich nanoparticles makes it possible to track ^19^F-labeled cells very selectively *in vivo* [5]. Fluorine is distinct from any other NMR (nuclear magnetic resonance)-active atom. Its negligible endogenous presence in the body provides essentially a ^19^F background free signal. As a consequence to this, as well as the possibility of overlaying cellular ^19^F images with anatomic ^1^H images,^ 19^F-MRI provides an important counterpart to ^1^H-MRI. The potential applications for ^19^F-rich compounds in magnetic resonance spectroscopy (MRS) and MRI have long been recognized [6]–[8]. Fluorine compounds that are commonly used in biomedical applications are chemically inert and synthetically derived perfluorocarbons (PFCs) that consist primarily of carbon and fluorine atoms. These fluorine-rich compounds are insoluble in water and must therefore be emulsified for clinically relevant applications such as intravenous, intraperitoneal or intraparenchymal injections. The particles obtained by emulsification typically have a size of approximately 200 nm. The size of the PFC particles employed in recent studies to label cells for *in vivo* ^19^F-MRI tracking ranged from 100 nm to 230 nm [5], [9]–[11]. Although nanotechnologists define nanoparticles as particles smaller than 100 nm [12], the definition is dynamic in the biological sciences, commonly referring to particles even up to 500 nm.

Increasing evidence suggests that the physico-chemical properties of nanoparticles determine the extent of their capacity to modulate the immune system [12]. In the present study we aimed to identify the outcome of changing the size of perfluoro-15-crown-5-ether (PFCE) particles on their uptake by dendritic cells (DC) and therefore the labeling efficiency, as well as on the impact on the immunological status of these cells. For this we prepared PFCE particles with different sizes ranging from 130 nm to 560 nm and incubated them with DC for ^19^F-labeling. To determine the impact of the particle size on ^19^F-labeling and immunomodulation, we performed MR spectroscopy and a battery of immunological tests, respectively. Our data show that the extent of DC ^19^F-labeling with 1mM PFCE was considerably improved upon increasing the particle size. At the same time we also show that an increase in PFCE particle size promotes the immunogenicity of DC.

## Results

### Perfluorocarbon particle size determines MR signal amplitude of DC

**Figure 1:**
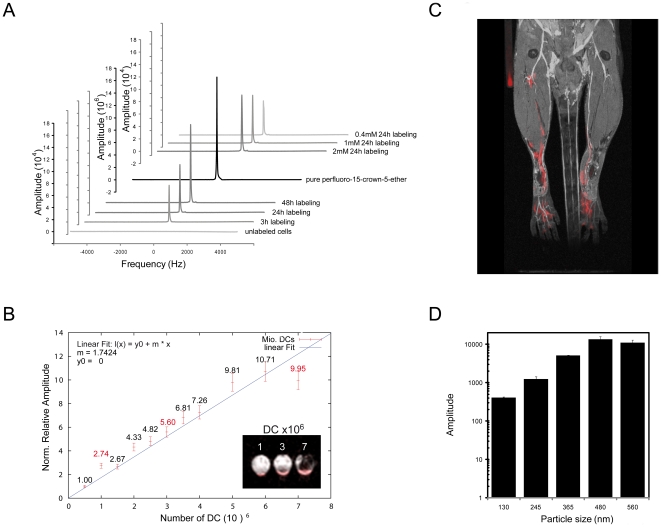
Perfluorocarbon particle size determines MR signal amplitude of DC. (A) DC were labeled with different concentrations and incubation times of 560 nm PFCE particles and their ^19^F signal was measured in a 3 T MRI scanner using a 90° block excitation pulse with 10 kHz bandwidth. (B) Different numbers of DC were labeled with 1 mM 560 nm PFCE particles over a period of 24 h and their ^19^F signal was measured in a 9.4 T MRI scanner. The inset depicts cross-sectional scans of the cell pellets within the NMR tubes made using a FLASH 3D Sequence on the 9.4 T scanner. (C) DC were labeled with 1 mM 560 nm PFCE particles, loaded with (right) or without (left) antigen and administered intradermally in hind limb. Shown is a coronal overlay of ^19^F cellular (red) and ^1^H anatomical (grayscale) MR images. (D) DC were labeled with different-sized particles and a constant PFCE concentration (1 mM) and their ^19^F signal amplitude was measured in a 9.4 T MRI scanner. This experiment is representative of 3 independent experiments

Using the *direct* sonication method we obtained particles with an average size of 245 nm±1.97 nm (±S.E.M.) that increased to 560 nm±6.36 nm following steam sterilization. The increase in particle size was also observed when we prepared PFCE particles using *high pressure homogenization*. Although particles doubled in size following steam sterilization, the size of particles achieved by both sonication and high pressure homogenization remained constant over several days under cell culture conditions (data not shown). Using the 560 nm particles, we labeled DC with different concentrations of PFCE together with different incubation times. The ^19^F signal amplitude was directly proportional to labeling time and PFCE concentration (**Fig. 1A**). The area under the curve (AUC) for DC samples incubated with particles containing 1 mM PFCE over a period of 3 h, 24 h and 48 h was calculated to be 9.28*10^7^, 1.43*10^8^ and 2.65*10^8^ respectively, while the AUC for DC samples incubated for 24 h with particles containing 0.4 mM and 2 mM PFCE was calculated to be 1.18*10^8^ and 2.22*10^8^ respectively. To extrapolate the ^19^F signal amplitude generated *in vitro* to the extent of DC localizing to specific regions *in vivo*, a calibration curve was made with different numbers of DC labeled with the same concentration of 560 nm particles (1 mM PFCE) and over a period of 24 h. The ^19^F signal amplitude (**Fig. 1B**) was calculated from ^19^F-MRS using the loop coil for the 9.4T MRI (**Supp. Fig. S1B**). The signal achieved was compared with the cellular *in vitro* cross-sectional images (FLASH 3D Sequence) of the ^19^F-labeled cell pellets within the NMR tubes (**Fig. 1B**, inset) using a commercial ^1^H/^19^F dual-tunable volume birdcage resonator for the 9.4T MRI (*see Methods*). The ^19^F signal amplitude to cell number curve gave a linear fit indicating that the signal of the spectroscopy data correlate linearly with the number of labeled cells (**Fig. 1B**). With the setup used the minimum amount of labeled cells detectable in MRS was 0.6*10^6^ DC corresponding to about 10^18^ fluorine atoms. For MRI this translates into a minimum cell number of 10^6^ DC within the volume of interest (**Fig. 1B**). Using the same concentration of 560 nm particles (1 mM PFCE) we could follow the migration of 10*10^6 19^F-labeled DC that were loaded with whole chicken ovalbumin (OVA) antigen. Following 18 h intradermal application of these cells in the right hind limb we observed a migration of the ^19^F-labeled cells from footpad into the draining popliteal lymph node, in contrast to cells that were not loaded with antigen and applied to the left hind limb (**Fig. 1C**). In the latter case the cells remained localized to the footpad.

The size of the PFC particles employed in other recent studies labeling cells for *in vivo* MRI tracking ranged from 100 nm to 230 nm [5], [9]–[11]. It is of profound importance to assess the impact of changes in the particle size on ^19^F signal amplitude. Hence we prepared an array of emulsions with different particle sizes ranging from 130 nm to 560 nm (**Table 1**). Figure 1D shows that larger particles with diameters of 365 nm, 480 nm and 560 nm provide a ^19^F signal of 5.1*10^3^, 1.3*10^4^ and 1.1*10^4^ arbitrary units, respectively, in comparison to the smaller particles with diameters of 130 nm and 150 nm that provide a ^19^F signal of 4.1*10^2^ and 1.2*10^3^ arbitrary units, respectively. Thus, an increase in particle size by a factor of approximately 4.3 (130 nm to 560 nm) results in an increase in signal by a factor of 26.8 (**Fig. 1D**). Furthermore the ^19^F signal appears to reach a plateau with particles larger than 480 nm.

**Table 1:** Particles size distribution of different perfluorocarbon emulsions.

Emulsion (particle size)	Z-Average Diam. (d.nm)	Peak Diam. (nm)	Peak Width (nm)	PdI
**130 nm**	130.1	139.0	36.67	0.056
**245 nm**	243.3	268.8	114.6	0.267
**365 nm**	367.3	404.7	127.2	0.091
**480 nm**	477.7	488.8	192.8	0.273
**560 nm**	565.7	533.4	122.6	0.179

For each emulsion we measured the following physical characteristics of the particles by using dynamic light scattering: Z-average diameter (mean diameter based on intensity of scattered light and sensitive to presence of large particles), peak diameter, peak width and polydispersity index (PdI) that gives a quantitative estimation of the particle size distribution.

### Morphological changes following uptake of perfluorocarbon particles

**Figure 2:**
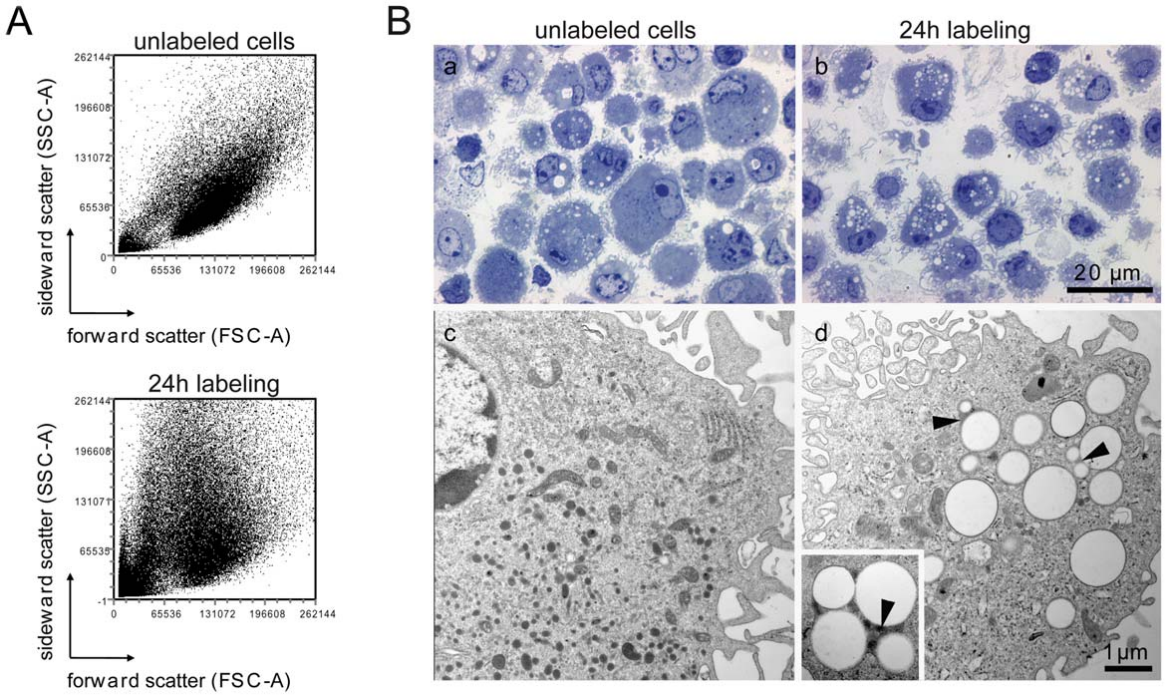
Perfluorocarbon particles increase DC granularity. (A) Forward and sideward scatter of unlabeled versus ^19^F-labeled DC as determined by FACS analysis. (B) Electron microscopy of DC pellets embedded in Poly/Bed 812 (see Methods). Morphology of DC in semithin (a,b) sections (x 100, bar = 20 µm) and ultrathin (c,d) sections (x 11 000, bar = 1 µm, inset×22 000). Arrowheads indicate vesicles with a membrane-like surface and an amorphous grey compartment.

Phagocytosis is a major component of the innate immune system since it is employed by professional antigen presenting cells (APC) such as DC to internalize particulate targets greater than 500 nm prior to initiation of the adaptive response [13]. Our initial data indicate that DC, as professional APC, take up larger particles more readily than smaller particles. Following uptake of the larger (560 nm) particles we also observed a significant increase in the granularity of DC, also discerned by an increase in the sideward light scatter (SSC-A) in FACS (**Fig. 2A**). Electron microscopy revealed the fluorine-rich particles as bright smooth spheroids (**Fig. 2B**) with a size ranging from about 180 nm to 1500 nm in diameter (average size = 660 nm±280 nm (S.E.M.), n = 150). Electron microscopy imaging of semithin sections of fixed DC pellets (**Fig. 2B a–b**) showed that most of the cells incubated for 24 h with 1mM PFCE particles (**Fig. 2B b**) contain clusters of vesicles in their cytoplasm. Imaging of ultrathin sections (**Fig. 2B c–d**) revealed that these vesicles in the nanoparticle-loaded DC (**Fig. 2B d**) have a membrane-like surface and an amorphous grey compartment (see also inset).

### Larger perfluorocarbon particles shift DC towards a mature status

**Figure 3:**
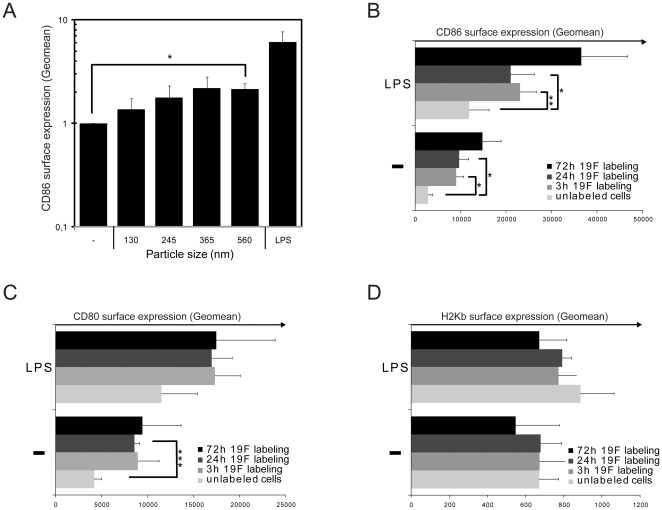
Larger perfluorocarbon particles shift DC towards a mature status. (A) Immature DC were labeled with different sizes of 1 mM PFCE particles. The surface CD86 expression (geometric mean _stained samples_–geometric mean _unstained controls_) was determined by FACS analysis. (B) Immature and LPS-matured DC were labeled with 1 mM PFCE particles (560 nm). The surface CD86, CD80 (C) and MHC/H2Kb (D) expression was determined as in *A*. Each figure represents the results of 3 grouped experiments.

The assessment of the direct impact of PFCE nanoparticles on the maturation status of DC provided different results for a spectrum of biomarkers. When we labeled DC with the same concentration of PFCE (1 mM) we observed a twofold increase in the surface expression of the co-stimulatory marker CD86 (as measured by FACS) on DC labeled with particles with a diameter of 560 nm (**Fig. 3A**). The increase in CD86 expression was time-dependent in both bacterial lipopolysaccharide (LPS)-treated and untreated (immature) cells (**Fig. 3B**). The increase in CD86 surface expression was accompanied by a significant increase in another B7 family member CD80 especially on immature DC (iDC) that had not been treated with LPS (**Fig. 3C**). The level of CD80 achieved following incubation of iDC with PFCE particles was comparable to that following triggering of Toll-like receptor 4 by LPS. We did not observe any changes in the surface expression of MHC (H2Kb) (**Fig. 3D**).

### Amplified T cell response by DC incubated with larger perfluorocarbon particles

**Figure 4:**
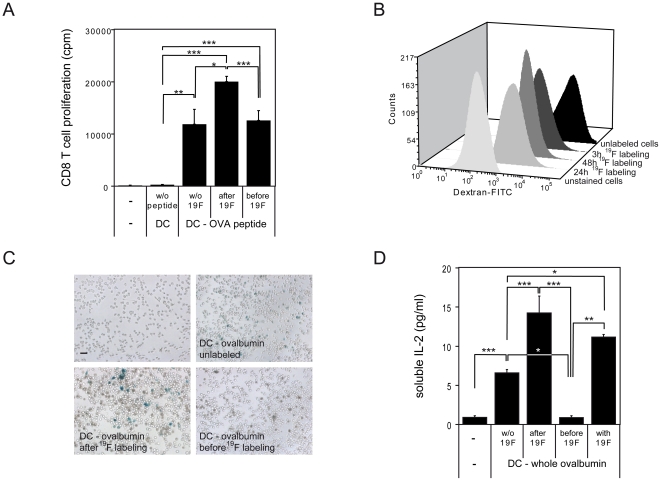
Larger perfluorocarbon particles hinder DC phagocytic activity but promote T cell response. (A) DC were incubated with/without ^19^F particles (560 nm) and ovalbumin (OVA) peptide and then co-incubated with naïve CD8^+^ T cells (CD8^+^CD44^-^CD62L^+^). Shown is the proliferation of CD8^+^ T cells measured in cpm (counts per minute) using a standard ^3^H-thymidine incorporation assay. This experiment is representative of 3 independent experiments. (B) DC were incubated with ^19^F particles (560 nm) over a period of time and their phagocytic capacity measured using a FITC-dextran incorporation assay. The fluorescence intensity of FITC-dextran in CD11c+ DC was measured by FACS analysis. This experiment is representative of 4 independent experiments. (C) DC were incubated with ^19^F particles (560 nm) and full-length endotoxin-free ovalbumin. Thereafter DC were co-incubated with B3Z T cells. Shown is an X-Gal staining of activated, β-galactosidase expressing (blue) B3Z T cells. This experiment is representative of 2 independent experiments. (D) DC were treated with ^19^F particles and full-length ovalbumin, thereafter co-incubated with B3Z T cells as in *C*. To quantify T cell activation, the amount of soluble IL-2 in the cell culture supernatants was measured by ELISA. This experiment is representative of 3 independent experiments.

CD86 (B7.2) provides the dominant costimulatory signal during early T cell activation [14]. Indeed an increased expression of CD86 on DC following treatment with the 560 nm PFCE particles was accompanied by an increase in priming of antigen-specific T cells by these DC (**Fig. 4A**). Naïve CD8^+^ T cells from C57BL/6-*Tg(OT-I)-RAG1^tm1Mom^* mice primed with DC loaded for 2 h with OVA peptide, 18 h following ^19^F labeling (CD86^hi^), proliferated significantly better than CD8^+^ T cells primed with unlabeled DC (*p*<0.05) or with DC that had been loaded with OVA peptide 2 h prior to ^19^F labeling (*p*<0.001) (**Fig. 4A**).

While mature DC may possess an increased capacity to stimulate T cells upon upregulation of costimulatory markers such as CD86, they are known to lose their capacity to take up antigen [15]–[18]. A recent study using particles larger than 500 nm also showed that particle size, in contrast to particle shape, influences phagocytosis completion [19]. Here our results demonstrate that DC labeled with 560 nm PFCE particles were less capable to phagocytize dextran as model antigen than unlabeled DC as shown by FACS staining (**Fig. 4B**). Dextran-FITC staining decreased progressively with prolonged labeling time (**Fig. 4B**).

An upregulation of maturation markers such as CD86 alone does not necessarily reflect an increased immunogenicity [20]. Considering that the ^19^F-labeled DC could not phagocytize optimally (**Fig. 4B**), we examined the impact of ^19^F labeling on the uptake of whole antigen by DC and presentation to T cells. As shown in **Fig. 4C**, ^19^F-labeled DC (brown) could present whole ovalbumin to and activate B3Z T cells even better than unlabeled DC when antigen was loaded after ^19^F labeling: more activated T cells (blue stain) are visible in this group than in the group consisting of T cells co-incubated with unlabeled DC or T cells co-incubated with DC that had been loaded with antigen prior to ^19^F labeling (**Fig. 4C**). The group of B3Z T cells activated by DC loaded with ovalbumin after ^19^F labeling secreted significantly more IL-2 than B3Z T cells that were co-incubated with unlabeled DC (*p*<0.001) or DC that had been loaded with ovalbumin prior to labeling with ^19^F nanoparticles (*p*<0.001) (**Fig. 4D**). These results together with the increased expression of costimulatory markers, as well as a decreased capacity of DC to phagocytize, suggest that the larger (560 nm) fluorine-rich particles do not obstruct with antigen presentation process but rather promote the transition of DC towards a mature status. Other than that, the particles seemed to be well-tolerated as shown by viability tests and apoptosis assays with AnnexinV/7-amino-actinomycin D (7-AAD) FACS staining (**Supp. Fig. S2**).

## Discussion

Fluorine MR imaging technology may pave the way towards clinical diagnostic and molecular/cellular imaging applications, particularly in immunologically related or driven diseases such as cancer, infection, autoimmunity or transplantation. Our data indicate that the larger fluorine-rich particles are taken up more readily by DC. An increase in particle size (despite a constant fluorine concentration) significantly enhanced the ^19^F-labeling of DC as shown by the increase in fluorine ^19^F spectral signal. Although this is favorable especially for the detection and imaging of smaller numbers of cells *in vivo*, the larger particles also precipitate concerns considering the changes in the immunological status that we observed in DC in this study.

We could show that the larger fluorine-rich particles promote the maturation process of DC and as a result the priming of naïve T cells. Notably, we observed that T cells were more actively primed by DC that were initially labeled with fluorine-rich particles (prior to peptide loading) than by DC that had been loaded with OVA peptide alone or OVA peptide (prior to ^19^F labeling). In the case of the OVA-specific CD8^+^ T cell hybridoma, we even observed a drastic reduction in T cell response when these cells were treated with DC loaded with full-length ovalbumin prior to ^19^F labeling. Future investigations on the timing of antigen-loading and nanoparticle labeling will help to further uncover interactions between antigen and ^19^F particles and the impact of their interaction on the delivery of stimulatory and costimulatory signals by DC to antigen-specific T cells.

From the present data, it seems that DC pretreated with fluorine-rich particles receive a maturation signal (CD86^hi^) and as a consequence present peptide more actively to naïve T cells. On the other hand, we think that DC initially incubated with antigen are unable to take up PFCE particles due to a down-regulation in uptake mechanisms. As a result these DC prime naïve T cells similarly as unlabeled DC and are even unable to activate antigen-specific responses as shown here with OVA_257-264_-specific T cells. A previous report on physicochemical properties of nanoparticles also demonstrated a skewing of the T cell response: particles larger than 1 µm induced a Th1 response in vivo, whereas particles smaller than 500 nm were associated with a Th2 response [21].

In our present study we presume that–in contrast to smaller particles (130 nm–365 nm)–the larger (560 nm) particles alter DC biology by precipitating different uptake mechanisms. Particles larger than 500 nm are commonly taken up by phagocytosis, in contrast to particles smaller than 500 nm that are usually taken up by endocytosis via structures such as caveolae or clathrin-coated vesicles [22]. However, apart from particle size, it is possible that other biologically-relevant changes in particle characteristics–such as shape and surface topology–could also alter DC biology. Scientists are indeed studying such physics-derived parameters to deliver solutions for controlling biological responses [23]. It would therefore be interesting to study the influence of these physical parameters (by changing surfactant or method of emulsification) on DC immunogenicity. Such studies are important since any undesirable modulation of the transplanted immune cells might pose potential hazards in certain clinical scenarios especially in those where the immune response should be kept under control e.g. in autoimmune disease and transplantation medicine. Thus, depending on the clinical situation in which *^19^*F-labeled cells are applied it will be important in the future to monitor the fate and impact of these cells *in vivo* using the relevant preclinical animal models. Even for cellular vaccines that are required to enhance the tumor-specific T cell response in cancer, care should be taken that the increased immunogenicity by ^19^F labeling does not precipitate severe or life-threatening autoimmunity.

## Materials and Methods

### Preparation of fluorine-rich particles

Particles with high fluorine (^19^F) content (100 mM–800 mM) were produced by emulsifying perfluoro-15-crown-5-ether (PFCE, Fluorochem, Derbyshire, UK), a chemically and biologically inert macrocycle consisting of 20 chemically equivalent fluorine (^19^F) nuclei, leading to only one signal in NMR spectra, which is important for quantification. PFCE was emulsified using Pluronic F-68 (Sigma-Aldrich, Germany) as emulsifying agent and by *direct sonication* with a cell disrupting titanium sonotrode (Bandelin Sonopuls GM70, Bandelin, Berlin, Germany). With this sonotrode we emulsified 200 mM PFCE in Pluronic F-68 by employing a continuous pulse program for 60 sec. Alternatively, an ultrasonic device connected to a block (*VialTweeter*, Hielscher Ultrasonics GmbH, Teltow, Germany) was used for *indirect* and more intense *sonication* of microtubes (1500 µl or 2000 µl) to reach the lower particle sizes. Different particle sizes were achieved by altering the sonication time and by changing the concentration of PFCE. We also employed *high pressure homogenization* (Microfluidizer® M-110S, Microfluidics, MA, USA) as an alternate method for emulsification, using pressures of up to 160 MPa. However, in contrast to the *sonication* method, *high pressure homogenization* required a minimum of 14 ml total volume for emulsification and sample losses of c. 10% were inevitable due to dead space. The average particle diameter was determined by dynamic light scattering using a Malvern Zetasizer Nano ZS instrument (Malvern Instruments, Worcestershire, UK). The physical characteristics of the particles generated with each new emulsion was documented and is shown in Table 1. Using both *direct* and *indirect* sonication systems it was possible to obtain particles with different sizes ranging from 130 nm to 560 nm. By autoclaving, particles generated by both *sonication* and *high pressure homogenization* increased to approximately double the original size. Since we employed these particles for DC immunological studies we tested endotoxin levels within all emulsions produced using the portable Endosafe®-PTS^TM^ (Charles River, Germany). We did not detect any endotoxins at a test sensitivity of 0.1 EU/ml.

### Generation of mouse bone-marrow (BM)-derived DC

DC were prepared from BM suspensions as previously described [24]. Briefly, BM from femurs of C57BL/6 mice were grown in RPMI-1640 medium containing 10% FCS (Biochrom, Germany) and supplemented with 10 ng/ml GM-CSF. For the analysis of maturation markers, cells were matured by incubating with 1 µg/ml lipopolysaccharide (LPS) for 24 h.

### In vitro MR-measurements

DC (3*10^6^) were labeled with emulsions containing different PFCE concentrations (0.4 mM, 1 mM, 2 mM), different particle sizes (130 nm–560 nm) and using different incubation times (3 h, 24, 48 h). Thereafter DC were thoroughly washed and fixed in 2% PFA. ^19^F uptake within fixed cells was monitored by ^19^F spectroscopy. Two separate ^19^F tuned loop coils (**Supp. Fig. S1A, 1B**) were designed for signal transmission and reception on a 3 Tesla (3 T) human MRI scanner (MedSpec 30/100, Bruker, Ettlingen, Germany) and a 9.4 T animal MRI scanner (Biospec 94/20-USR, Bruker, Ettlingen, Germany). The ^19^F signal was acquired using a 90° block excitation pulse with 10 kHz bandwidth and the amplitude was calculated by performing a fast Fourier transformation (FFT) of the acquired free induction decay (FID). Alternatively, the area under the curve (AUC) of the spectra was calculated. For comparison, ^19^F baseline MR spectra were acquired prior to labeling.

### In vivo MR-measurements

Animal experiments were carried out in accordance with the guidelines provided and approved by the Animal Welfare Department of the *LAGeSo* State Office of Health and Social Affairs Berlin (Permit G0070/09: *Migration v. Immunzelltherapien*, 15.07.2009-31.07.2012). Prior to *in vivo* application, DC were incubated for 24 h with ^19^F emulsion (using a concentration of 1 mM perfluoro-15-crown-5-ether and 560 nm particle size) and 6 h with 5 µg/ml full-length chicken EndoGrade ovalbumin (endotoxin conc.<1 EU/mg; Hyglos, Regensburg, Germany). Thereafter, DC were harvested, washed thoroughly in serum-free buffer, administered intradermally (3–10*10^6^) into the left hind limb of C57BL/6 mice and imaged 18 h following injection. Shortly before and during the MR session, mice were anesthetized using a mixture of isoflurane as inhalation narcosis (0.5–1.5%), pressurized air and oxygen. Mice were imaged on the 9.4 T animal MRI using a custom made ^1^H/^19^F dual-tunable volume birdcage resonator (Rapid Biomed, Würzburg, Germany) with 35 mm inner diameter and 50 mm length. The temperature of the mice was regulated at 37°C. The breathing rate and temperature was monitored by a remote monitoring system (Model 1025, SA Instruments Inc., New York, USA). Images were acquired using a 3D-FLASH gradient-echo sequence. For ^19^F imaging, TR = 8 ms; TE = 3 ms; flip angle = 10°; FOV = 50×25×25 mm, matrix = 200×100×100; number of averages = 80. For ^1^H imaging, TR = 11 ms; TE = 4 ms; flip angle = 15°; FOV = 50×25×25 mm; matrix = 400×200×200, number of averages = 8. For both *in vivo* and *in vitro* MR-measurements a Redhat RHEL4 system and Paravision v5 (Bruker Biospin, Ettlingen, Germany) software were used.

### Electron microscopy

Cells were fixed for 24 hours in PBS containing 2% glutaraldehyde and postfixed for 2 h with 1% osmium tetroxide. Cell pellets were then dehydrated in an ascending series of ethanol and embedded in Poly/Bed 812 (Polysciences, Eppelheim, Germany). Semithin sections of the DC pellets were stained with toluidine blue. Ultrathin sections were stained with uranyl acetate and lead citrate. Sections were imaged using a FEI Morgagni electron microscope (FEI, Eindhoven, The Netherlands) and iTEM software.

### Annexin/7-AAD assay

Harvested iDC were washed with ice cold PBS and resuspended in binding buffer (10 mM Hepes, pH 7.4, 140 mM NaCl, 2.5 mM CaCl_2_). Thereafter, 10^5^ cells were incubated with 7-amino-actinomycin D (7-AAD) and PE-conjugated annexinV (BD Biosciences, Heidelberg, Germany), and then analyzed by FACS using a FACSCanto II flow cytometer equipped with FACSDiva software (BD Biosciences, Heidelberg, Germany). The percentages of AnnexinV^-^/7-AAD^-^, AnnexinV^+^/7-AAD^-^, and AnnexinV^+^/7-AAD^+^ populations were calculated corresponding to the distribution in the set quadrants.

### Surface staining of costimulatory molecules and MHC-I

To determine the maturation status, DC were washed with FACS buffer, stained with anti-CD80, anti-CD86 and MHC-I (H2Kb) antibodies and analyzed by FACS using a FACSCanto II and FACSDiva software (BD Biosciences, Heidelberg, Germany).

### T cell priming

Naïve CD8^+^ T cells (CD8^+^CD44^-^CD62L^+^) were sorted from spleen and LN cells of C57BL/6-*Tg(OT-I)-RAG1^tm1Mom^* mice (Taconic, Lille Skensved, Denmark) using FACS sorting (FACSAria, BD Biosciences). *Tg(OT-I)-RAG1^tm1Mom^* mice are homozygous for a transgene encoding, a T cell receptor specific for OVA_257–264_ presented by the MHC class I molecule H-2Kb. iDC were incubated for 2 h with 10 µg/ml ovalbumin peptide (OVA_257–264_) and thereafter incubated with sorted naïve CD8^+^ T cells. T cell proliferation (as a measure of T cell priming) was determined 3 d later using a ^3^H thymidine incorporation assay (see below). For experiments where DC were labeled with PFCE particles, OVA peptide was given either prior to or following PFCE labeling: iDC were either incubated for 18 h with PFCE particles and thereafter for an additional 2 h with OVA_257–264_ prior to incubation with naïve T cells (DC–OVA peptide after 19F), or alternatively incubated for 2 h OVA_257–264_ and thereafter 18 h with PFCE particles prior to incubation with naïve T cells (DC–OVA peptide before 19F).

### ^3^H thymidine incorporation proliferation assay

[^3^H]thymidine (0.5 µCi) (Amersham Biosciences, Freiburg, Germany) was added to each well of a proliferation assay plate. Incorporation of radioactivity was measured after 18 h with a TopCount NXT beta counter (PerkinElmer LAS GmbH, Rodgau-Jügesheim, Germany).

### FITC-dextran incorporation assay

iDC (1×10^5^) were resuspended in 100 µl PBS and incubated with 1 mg/ml FITC-dextran (Sigma-Aldrich Chemie, Schnelldorf, Germany) at 37°C and 0°C (negative control) for 30 min. The incubations were stopped by adding 2 ml of ice-cold PBS containing 1% serum and 0.02% sodium azide. After thorough washing, cells were analyzed by FACS (FACSCanto II). Appropriate gates were made on viable CD11c+ cells to exclude debris and dead cells.

### Activation of B3Z cells

DC were loaded with 5 µg/ml full-length chicken EndoGrade ovalbumin (endotoxin conc.<1 EU/mg; Hyglos, Regensburg, Germany) for 18 h and thereafter co-incubated with B3Z cells that express a TCR specifically recognizing OVA_257–264_ and carry a β-galactosidase (lacZ) construct driven by nuclear factor of activated T cells elements from the IL-2 promoter [25]. For experiments where DC were labeled with PFCE particles, full-length ovalbumin was given either prior to or following PFCE labeling as described above (*T cell priming*) for OVA_257–264_ peptide loaded DC. To determine the activation status of B3Z T cells, intracellular β-galactosidase activity was measured by X-Gal staining as previously described [26]. Briefly, cells were fixed in 0.05% glutaraldehyde and incubated for 24 h at 37°C in X-Gal staining solution.

### Measurement of soluble IL-2

Supernatants were collected 24 h following co-culturing DC with B3Z cells and stored at −80°C. Soluble IL-2 was measured by ELISA following the company's instructions (eBioscience, NatuTec, Frankfurt, Germany).

### Statistics

Statistical analysis was performed with SPSS 12 (SPSS Inc., Chicago, IL, USA). For group comparisons, the t-test was used *: p-value<0.05, **<0.01, ***<0,001. Graphical presentation was done with SigmaPlot 10 (Systat Software, Germany), Excel ® 2007 (Microsoft ® Office) and Gnuplot v4.2.6 by T. Williams and C. Kelly (http://www.gnuplot.info/). All data presented in the bar graphs indicate the mean and the standard error of the mean (S.E.M.).

## Supporting Information

Figure S1 **Design of ^19^F loop coils for ^19^F-MRS.** (A) Loop coil (5-turn) that holds NMR-tubes for measuring ^19^F signal in cell pellets using a human 3 T scanner (B) Loop coil (4-turn) for measurement of ^19^F-labeled cells in NMR-tubes using an animal 9.4 T scanner.
(TIF)

Figure S2 **Perfluorocarbon particles are well tolerated by DC.** DC were incubated with PFCE particles (560 nm) over a period of 3 days after which cells were stained with AnnexinV-PE and 7-AAD and measured by FACS. Depicted in each quadrant is the percentage of cell populations in different conditions: viable (LL, AnnexinV^-^, 7-AAD^-^), early apoptotic (LR, AnnexinV^+^, 7-AAD^-^), late apoptotic or dead (UR, AnnexinV^+^/7-AAD^+^), dead (UL, AnnexinV^-^, 7-AAD^+^).
(TIF)

## References

[1] Banchereau J, Schuler-Thurner B, Palucka AK, Schuler G (2001). Dendritic cells as vectors for therapy.. Cell.

[2] Mackensen A, Krause T, Blum U, Uhrmeister P, Mertelsmann R (1999). Homing of intravenously and intralymphatically injected human dendritic cells generated in vitro from CD34+ hematopoietic progenitor cells.. Cancer Immunol Immunother.

[3] de Vries IJ, Lesterhuis WJ, Barentsz JO, Verdijk P, van Krieken JH (2005). Magnetic resonance tracking of dendritic cells in melanoma patients for monitoring of cellular therapy.. Nat Biotechnol.

[4] Brooks RA, Brunetti A, Alger JR, Di Chiro G (1989). On the origin of paramagnetic inhomogeneity effects in blood.. Magn Reson Med.

[5] Ahrens ET, Flores R, Xu H, Morel PA (2005). In vivo imaging platform for tracking immunotherapeutic cells.. Nat Biotechnol.

[6] Lauterbur PC (1973). Image Formation by Induced Local Interactions - Examples Employing Nuclear Magnetic-Resonance.. Nature.

[7] Holland GN, Bottomley PA, Hinshaw WS (1977). F-19 Magnetic-Resonance Imaging.. Journal of Magnetic Resonance.

[8] Liu MS, Long DM (1977). Perfluoroctylbromide as a diagnostic contrast medium in gastroenterography.. Radiology.

[9] Partlow KC, Chen J, Brant JA, Neubauer AM, Meyerrose TE (2007). 19F magnetic resonance imaging for stem/progenitor cell tracking with multiple unique perfluorocarbon nanobeacons.. FASEB J.

[10] Ruiz-Cabello J, Walczak P, Kedziorek DA, Chacko VP, Schmieder AH (2008). In vivo “hot spot” MR imaging of neural stem cells using fluorinated nanoparticles.. Magn Reson Med.

[11] Srinivas M, Morel PA, Ernst LA, Laidlaw DH, Ahrens ET (2007). Fluorine-19 MRI for visualization and quantification of cell migration in a diabetes model.. Magn Reson Med.

[12] Dobrovolskaia MA, McNeil SE (2007). Immunological properties of engineered nanomaterials.. Nat Nanotechnol.

[13] Aderem A, Underhill DM (1999). Mechanisms of phagocytosis in macrophages.. Annu Rev Immunol.

[14] Borriello F, Sethna MP, Boyd SD, Schweitzer AN, Tivol EA (1997). B7-1 and B7-2 have overlapping, critical roles in immunoglobulin class switching and germinal center formation.. Immunity.

[15] Austyn JM, Kupiec-Weglinski JW, Hankins DF, Morris PJ (1988). Migration patterns of dendritic cells in the mouse. Homing to T cell-dependent areas of spleen, and binding within marginal zone.. J Exp Med.

[16] Sallusto F, Lanzavecchia A (1994). Efficient presentation of soluble antigen by cultured human dendritic cells is maintained by granulocyte/macrophage colony-stimulating factor plus interleukin 4 and downregulated by tumor necrosis factor alpha.. J Exp Med.

[17] Winzler C, Rovere P, Rescigno M, Granucci F, Penna G (1997). Maturation stages of mouse dendritic cells in growth factor-dependent long-term cultures.. J Exp Med.

[18] Roake JA, Rao AS, Morris PJ, Larsen CP, Hankins DF (1995). Dendritic cell loss from nonlymphoid tissues after systemic administration of lipopolysaccharide, tumor necrosis factor, and interleukin 1.. J Exp Med.

[19] Champion JA, Mitragotri S (2006). Role of target geometry in phagocytosis.. Proc Natl Acad Sci U S A.

[20] Menges M, Rossner S, Voigtlander C, Schindler H, Kukutsch NA (2002). Repetitive injections of dendritic cells matured with tumor necrosis factor alpha induce antigen-specific protection of mice from autoimmunity.. J Exp Med.

[21] van Zijverden M, Granum B (2000). Adjuvant activity of particulate pollutants in different mouse models.. Toxicology.

[22] Pelkmans L (2005). Secrets of caveolae- and lipid raft-mediated endocytosis revealed by mammalian viruses.. Biochim Biophys Acta.

[23] Mitragotri S, Lahann J (2009). Physical approaches to biomaterial design.. Nat Mater.

[24] Bendix I, Pfueller CF, Leuenberger T, Glezeva N, Siffrin V (2010). MAPK3 deficiency drives autoimmunity via DC arming..

[25] Shastri N, Gonzalez F (1993). Endogenous generation and presentation of the ovalbumin peptide/Kb complex to T cells.. J Immunol.

[26] Sommermeyer D, Neudorfer J, Weinhold M, Leisegang M, Engels B (2006). Designer T cells by T cell receptor replacement.. Eur J Immunol.

