# Supplementary figures and images for: Perfluorocarbon Particle Size Influences Magnetic Resonance Signal and Immunological Properties of Dendritic Cells
Source: PLoS One. 2011 Jul 19;6(7):e21981. doi: 10.1371/journal.pone.0021981 (PMC3139612; doi:10.1371/journal.pone.0021981)

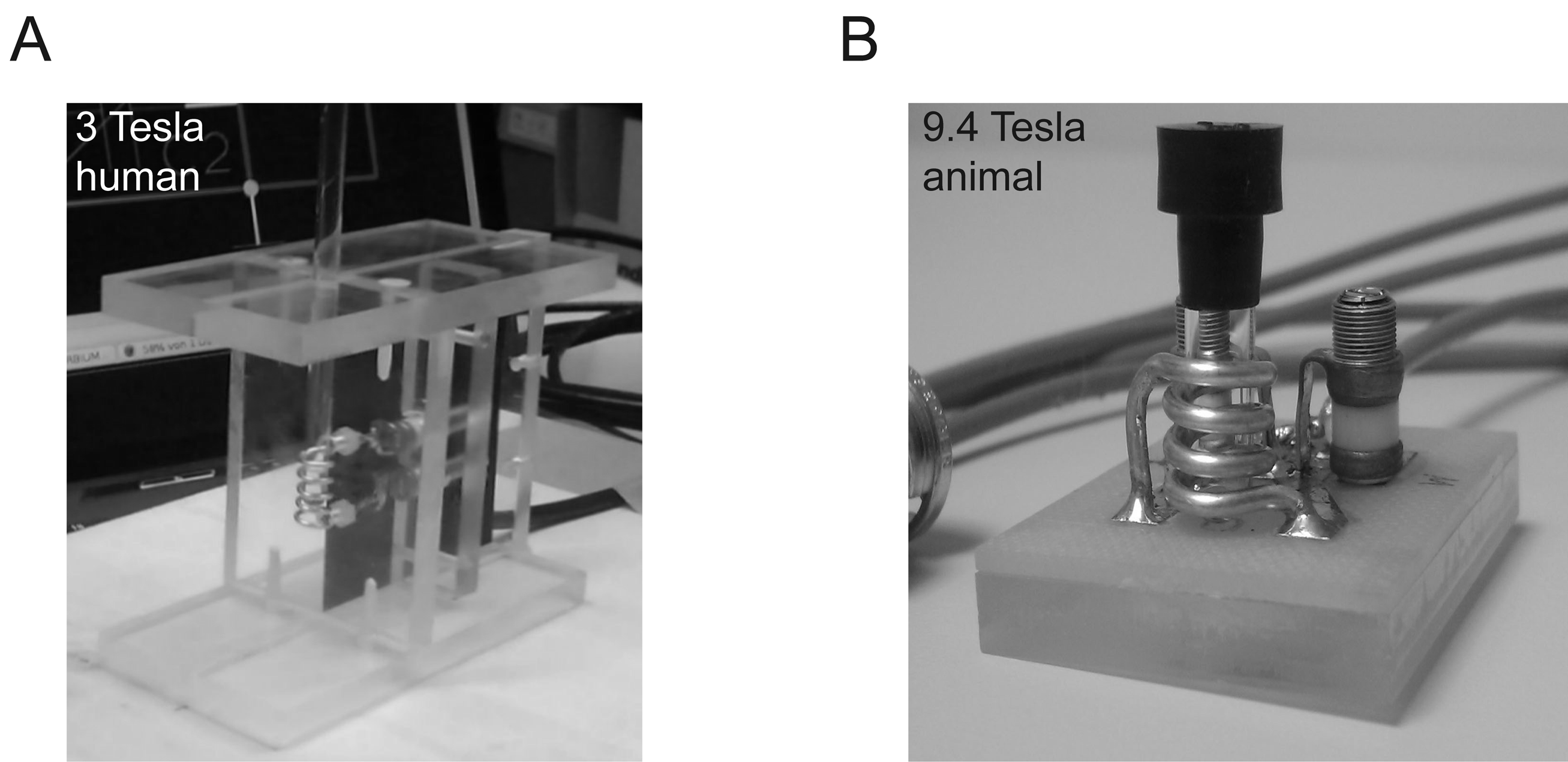

Supplement: Figure S1 — Design of 19F loop coils for 19F-MRS. (A) Loop coil (5-turn) that holds NMR-tubes for measuring 19F signal in cell pellets using a human 3 T scanner (B) Loop coil (4-turn) for measurement of 19F-labeled cells in NMR-tubes using an animal 9.4 T scanner. (TIF) [file pone.0021981.s001.tif]

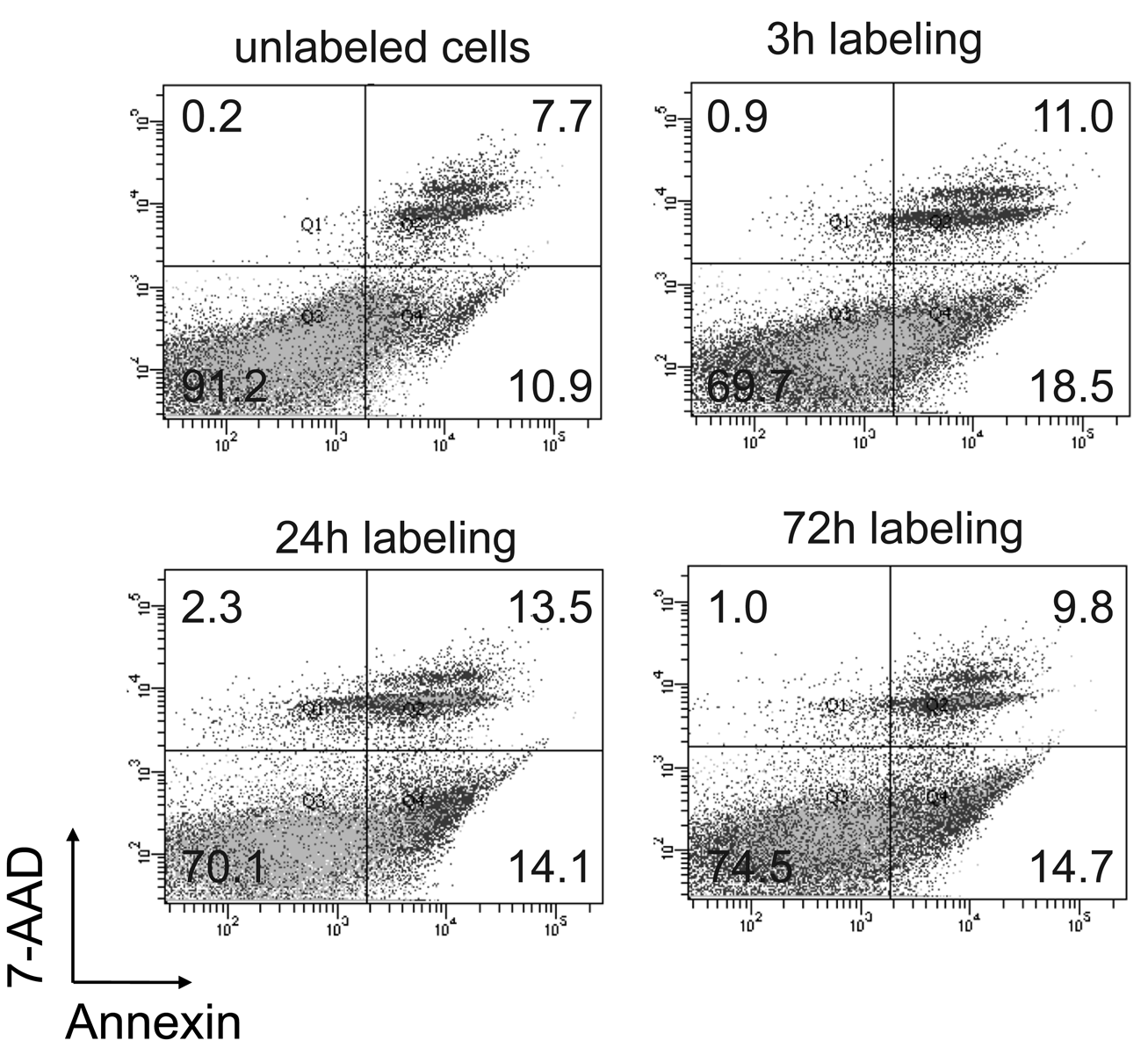

Supplement: Figure S2 — Perfluorocarbon particles are well tolerated by DC. DC were incubated with PFCE particles (560 nm) over a period of 3 days after which cells were stained with AnnexinV-PE and 7-AAD and measured by FACS. Depicted in each quadrant is the percentage of cell populations in different conditions: viable (LL, AnnexinV-, 7-AAD-), early apoptotic (LR, AnnexinV+, 7-AAD-), late apoptotic or dead (UR, AnnexinV+/7-AAD+), dead (UL, AnnexinV-, 7-AAD+). (TIF) [file pone.0021981.s002.tif]
